# An Antibody Specific for the Dog Leukocyte Antigen DR (DLA-DR) and Its Novel Methotrexate Conjugate Inhibit the Growth of Canine B Cell Lymphoma

**DOI:** 10.3390/cancers11101438

**Published:** 2019-09-26

**Authors:** Marta Lisowska, Magdalena Milczarek, Jarosław Ciekot, Justyna Kutkowska, Wojciech Hildebrand, Andrzej Rapak, Arkadiusz Miazek

**Affiliations:** 1 Department of Tumor Immunology, Hirszfeld Institute of Immunology and Experimental Therapy, Polish Academy of Sciences, 53-114 Wroclaw, Poland; marta.lisowska@hirszfeld.pl; 2 Department of Experimental Oncology, Hirszfeld Institute of Immunology and Experimental Therapy, Polish Academy of Sciences, 53-114 Wroclaw, Poland; magdalena.milczarek@hirszfeld.pl (M.M.); jaroslaw.ciekot@hirszfeld.pl (J.C.); justyna.kutkowska@hirszfeld.pl (J.K.); 3 Veterinary Clinic NeoVet, 52-225 Wroclaw, Poland; hildek@interia.eu; 4 Department of Biochemistry and Molecular Biology, Wroclaw University of Environmental and Life Sciences, 50-375 Wroclaw, Poland; 5 Centre of Genetic Engineering, Wroclaw University of Environmental and Life Sciences, 50-366 Wroclaw, Poland

**Keywords:** passive immunotherapy, canine B-cell lymphoma, DLA-DR, HLA-DR, antibody-drug conjugate, ADC, methotrexate

## Abstract

Canine B-cell lymphoma (CBL) is an incurable, spontaneous lymphoid malignancy constituting an accurate animal model for testing novel therapeutic strategies in human medicine. Resources of available species-specific therapeutic monoclonal antibodies (mAbs) targeting CBL are scarce. The aim of the present study was to evaluate the therapeutic potential of mAb B5, specific for the dog leukocyte antigen DR (DLA-DR) and its antibody-drug conjugate with methotrexate (B5-MTX). B5 induced caspase-dependent apoptosis of DLA-DR-expressing canine B cell lymphoma/CLBL1 and CLB70 leukemia lines, but not the GL-1 line not expressing DLA-DR. The cytotoxicity of B5-MTX to sensitive cells was further potentiated by a payload of MTX, but without any substantial off-target effects. The infusion of B5 and B5-MTX in a murine model of disseminated, advanced canine lymphoma, mediated >80% and >90% improvement in survival, respectively, and was well tolerated by the animals. Interestingly, the concentrations of soluble DLA-DR (sDLA-DR) antigens present in the blood serum of tumor-bearing mice were found proportional to the tumor burden. On this basis, sDLA-DR levels were evaluated as a potential biomarker using samples from canine lymphoma patients. In summary, the action of B5 and B5-MTX holds promise for further development as an alternative/complementary option for the diagnosis and treatment of canine lymphoma.

## 1. Introduction

Canine B cell lymphoma (CBL) is a spontaneous malignancy bearing numerous molecular, histopathological and clinical similarities to human non-Hodgkin lymphoma (NHL) [1]. For this reason, dogs are considered an important animal model for pre-clinical testing of new therapies for human lymphoma [2,3]. CBL is the most frequent hematological malignancy with various histopathological presentations and accounts for over 60% of all diagnosed lymphoma cases in dogs [4]. Around 16,000 to 80,000 dogs owned in the United States alone suffer from hematological malignancies annually [5,6]. Current clinical management of CBL involves combination chemotherapy, but in contrast to human regimens, it employs lower doses of cytostatics and lacks biologicals. Relapses of the disease are usually observed within 10–14 months post-treatment, with less than 25% of dogs surviving two years [2].

The use of a therapeutic anti-CD20 monoclonal antibody (rituximab) has greatly ameliorated NHL treatment, but direct application of rituximab for CBL treatment is impossible due to the lack of amino acid sequence conservation between human and canine CD20 [7]. Efforts to raise therapeutic monoclonal antibodies to canine CD20 have resulted in the development of several candidate reagents [8,9,10]. Of those, 1E4 and its caninized derivatives showed a therapeutic effect against the CLBL1 canine lymphoma cell line in vitro and in vivo [11].

Major histocompatibility antigen class II antigen DR (MHC II DR) is an attractive and alternative target to CD20 for passive immunotherapy of NHL and CBL [12]. MHC II DR is highly expressed by B cell neoplasms in humans and dogs with mean cell surface levels exceeding those of CD20 [13,14]. Research on mAbs targeting MHC II DR dates back to 1987, when eradication of murine lymphoma in a syngeneic model stimulated the development of similar human strategies [15]. However, a record of limited success in clinical trials and safety concerns related to immune toxicity raised some doubts about further exploration of these therapeutic mAbs [16]. The renaissance of interest in therapeutic HLA-DR targeting came with the characterization of the humanized murine L243 antibody, named IMMU-114, which is specific for a monomorphic determinant on the HLA-DR alpha chain [12]. In preclinical trials, it demonstrated a better efficacy in killing various hematological malignancies than CD20. Moreover, it displayed a promising efficacy in phase I clinical trial in relapsed or refractory NHL and in chronic lymphocytic leukemia (CLL) [17]. With the advent of antibody-drug conjugate (ADC) technology, IMMU-114 has recently been modified to carry a payload of an active irinotecan metabolite. With this payload, therapeutic effects were observed in preclinical models of IMMU-114-resistant tumors such as acute myeloblastic leukemia and malignant melanoma [18]. A good safety profile of IMMU-114 has been reported both in human and canine patients [14]. However, due to the limited cross-reactivity of IMMU-114 with canine DLA-DR [19], a thorough assessment of the full therapeutic potential of this target in dogs is difficult.

In the search for species-specific mAbs for therapeutic targeting of DLA-DR, we have developed a murine mAb, B5. This antibody binds strongly to a conformational epitope of canine DR alpha chain (DLA-DRα), but shows only minimal cross-reactivity with HLA-DRα. We have previously shown that B5 exerts immune-dependent and direct cytotoxic effects in vitro [19].

Here, we extend these studies and report on the generation and pre-clinical testing of the novel B5 ADC with methotrexate (B5-MTX). Methotrexate (MTX) is an inexpensive and pharmacologically well-characterized antimetabolite drug [20]. Canine lymphoma cell lines were found to be 10 times more sensitive to MTX (IC50 values of 2–3 nM) than the human Raji B cell lymphoma and Jurkat T-ALL cell lines [21]. At high doses, MTX is still used in combination with rituximab to treat Burkitt’s lymphoma and primary mediastinal B-cell lymphoma [22]. Conjugation of mAbs with MTX changes their mode of entry to target cells [23]. It has been shown in several studies that the MTX payload increases the rate of tumor cell inhibition due to rapid conjugate uptake and an increase in sensitivity to direct cytotoxic effects of therapeutic mAbs [23]. We hypothesized that conjugating mAb B5 with MTX can exert an additive effect of both components and contribute to a better cytotoxicity profile of this ADC against CBL.

We report as well on a novel enzyme-linked immunosorbent assay (ELISA) for the detection of circulating soluble DLA-DR (sDLA-DR) complexes in the blood serum of dogs. Physiologically, soluble circulating MHC II molecules (sMHC II) loaded with self-peptides contribute to the maintenance of self-tolerance [24]. They can be released from antigen-presenting cells or tumor B cells as well and suppress T cell immune-surveillance by directly competing with membrane-bound MHC II ligands [24]. In this report, we aimed at testing the hypothesis that blood serum levels of sDLA-DR could be indicative of tumor burden. We present observations supporting sDLA-DR as a potentially useful biomarker for monitoring the outcome of CBL chemotherapy. Overall, our data indicate the potential therapeutic and diagnostic value of anti-DLA-DR-specific antibodies in CBL.

## 2. Results

### 2.1. Characterization of the B5-MTX Conjugate

**Figure 1:**
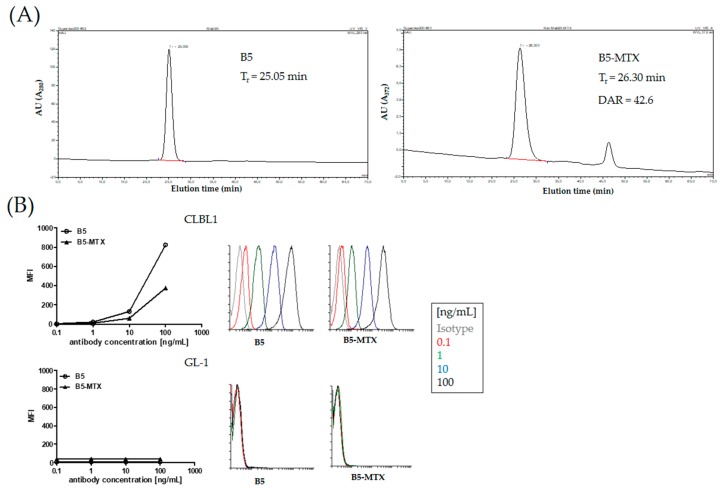
Synthesis of B5-MTX ADC. (**A**) Size-exclusion HPLC of unmodified mAb B5 (top), detected at A280 nm and B5-MTX conjugate (bottom) detected at A372 nm (peak elution absorbance is given in absorbance units—AU) with a molar ratio of MTX to mAb (DAR) of 42.6. The conjugate was >94% pure and monomeric. Retention time (tr) difference of free mAb and B5-MTX resulted from the high substitution rate. (**B**) Flow cytometry assessment of B5 and B5-MTX staining intensity (MFI-mean fluorescence intensity) in DLA-DR expressing CLBL1 and non-expressing GL-1 cell lines. Isotype control mouse IgG2a antibody was used at the concentration of 100ng/mL (grey histogram). Color histograms correspond to signal intensities obtained with the indicated concentrations of antibody or conjugate in [ng/mL].

Crosslinking of MTX anhydride with mAb B5 resulted in >94% homogenous preparation of the B5-MTX antibody-drug conjugate (Figure 1A). Size-exclusion HPLC analysis of unmodified mAb B5 versus B5-MTX revealed a delay in retention times (tr = 25.05 versus tr = 26.3), which was due to the high average drug to antibody substitution ratio (DAR), estimated at 42.6 (Figure 1A). The visible second peak at retention time tr = 46.317 min corresponded to free MTX dissociated from the conjugate. The resulting B5-MTX conjugate demonstrated approximately 49% loss of target binding activity in comparison to unconjugated B5 and had negligible nonspecific binding activity (Figure 1B).

### 2.2. Cytotoxicity of B5 and B5-MTX Against Canine Lymphoma/Leukemia Lines In Vitro

To evaluate the cytotoxicity of B5 and B5-MTX, canine B cell lymphoma/leukemia cell lines expressing DLA-DR (CLBL1 and CLB70) and not expressing DLA-DR (GL-1) were exposed to 2 μg/mL of both preparations for 48 h. As previously reported [19], several hallmarks of direct apoptotic cell death, including caspase 3/7 activation, annexin V binding, DNA fragmentation (subG1 DNA content), were observed upon B5 treatment of DLA-DR expressing cell lines (Supplementary Figure S1). In comparison with B5, the B5-MTX conjugate exerted more potency, but also cell-specific cytotoxic effects at the same time, as no significant increase in toxicity against the DLA-DR non-expressing GL-1 cell line was detected. The average percentages of cell death induced by several tested concentrations (0.1–10 μg/mL) of B5 and B5-MTX were used to calculate IC50 and maximum inhibition values of both preparations. B5-MTX showed a higher maximum cytotoxicity (85% to 88% versus 65% to 69%) and lower IC50 values (5–6.25 nM versus 9.53–11.5 nM) against the CLBL1 and CLB70 cell lines than B5 (Table 1).

**Figure 2:**
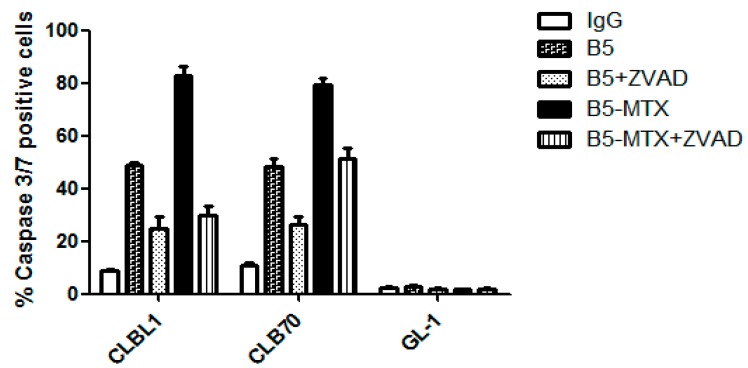
Assessment of caspase 3/7 activation after treatment of individual canine cell lines with B5 and B5-MTX in the presence or absence of a pan-caspase inhibitor (ZVAD). The average percentages of caspase 3/7-activating cells with ±SD were calculated from at least two independent experiments. Every sample was assessed in triplicate.

Reportedly, MHC II cross-linking can trigger either caspase-dependent or caspase-independent cell death mechanisms [12]. In order to determine whether B5- and B5-MTX-induced apoptosis was caspase-dependent, cell lines indicated in Figure 2 were pre-incubated with a pan-caspase inhibitor, ZVAD, prior to treatment with individual antibody preparations. On average, a 50% decrease in caspase 3/7 activating cells was detected after ZVAD pretreatment of CLBL1 and CLB70 cells, but without unspecific effects on the GL-1 cell line. Inhibition of apoptosis by ZVAD was minimally lower for the CLB70 cell line treated with B5-MTX than for the similarly treated CLBL1 cell line.

Together, these data indicated that both B5 and B5-MTX displayed a potent caspase-dependent anti-tumor activity against the CLBL1 and CLB70 cell lines, but not against the GL-1 line in vitro. The MTX payload carried by the B5-MTX conjugate enhanced the specific cytotoxicity compared to B5 alone. The ability of a pan-caspase inhibitor, ZVAD, to strongly interfere with B5- and B5-MTX-induced cell killing supported the caspase-dependent mechanism.

### 2.3. Therapeutic Efficacy of B5 and B5-MTX in NOD-SCID Mice Xenotransplanted with the CLBL1-Luc Cell Line

**Figure 3:**
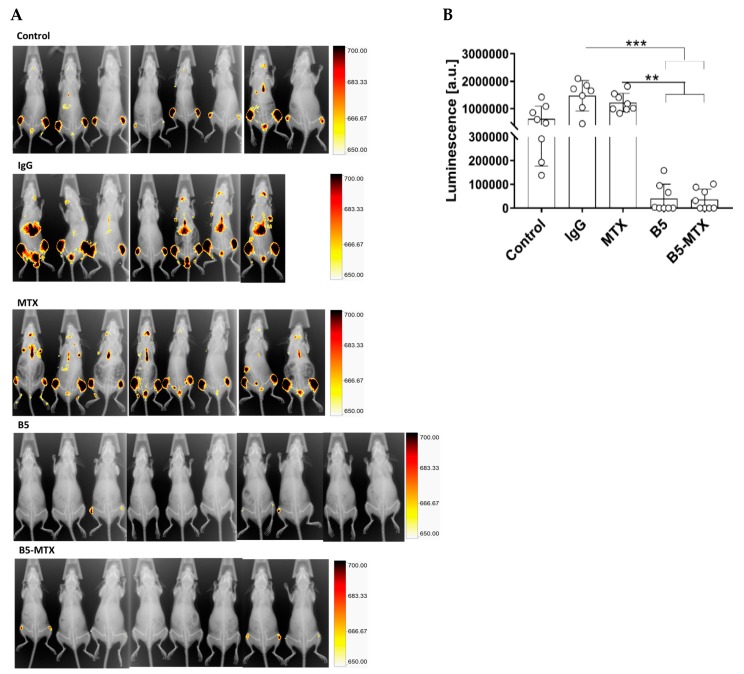
In vivo imaging and quantification of bioluminescence in CLBL1-Luc tumor-bearing mice on day 15 after tumor cell transplantation. (**A**) Control mice were infused with phosphate-buffered saline (Control), isotype-matched mouse IgG immunoglobulin (IgG), free methotrexate (MTX) or were treated with mAb B5 and B5-MTX. Bioluminescence intensity is presented on pseudo-color scales. (†) The IgG group contained seven mice because one mouse was found dead on the day preceding bioluminescence imaging (B) Individual bioluminescence intensities of each mouse were plotted. Statistically significant differences between the groups were marked with an asterisk (*** *p* < 0.001, ** *p* < 0.01).

To evaluate the efficacy of B5 and B5-MTX treatment in vivo, we established a disseminated disease model in which 1 × 10^7^ cells of the luciferase-expressing CLBL1-derived cell line (CLBL1-Luc) were injected intravenously into immune-deficient NOD-SCID mice. A total of 42 animals were randomly assigned to five groups treated as follows: PBS (*n* = 8), IgG (*n* = 8), MTX (*n* = 8), B5 (*n* = 10), B5-MTX (*n* = 8). Four days after the CLBL1-Luc injection, mice were treated three times a week. On day 15, all mice in PBS, IgG and MTX treatments were sacrificed because of weight loss and signs of morbidity. Five randomly selected mice from the B5 group and five mice from the B5-MTX group, showing no visible symptoms of health deterioration, were sacrificed along with control mice. Blood and organs from sacrificed mice were further analyzed as described below. The remaining animals were treated with B5 and B5-MTX until day 20 and sacrificed once their weight loss exceeded 15% or when they became moribund. On day 15 post-CLBL1-Luc injection, bioluminescence imaging was performed to assess tumor burden. Foci of intense tumor growth in the groups of control mice were located mostly in hind limb bones and in some distant organs. In B5 and B5-MTX treated mice, tumor growth was only localized in the hind limbs of certain mice, whereas other mice had virtually no signs of localized tumor growth (Figure 3A). The intensities of individual bioluminescence measurements for each mouse are plotted in Figure 3B. Groups treated with B5 and B5-MTX presented at least 20 times lower signal intensities than controls.

**Figure 4:**
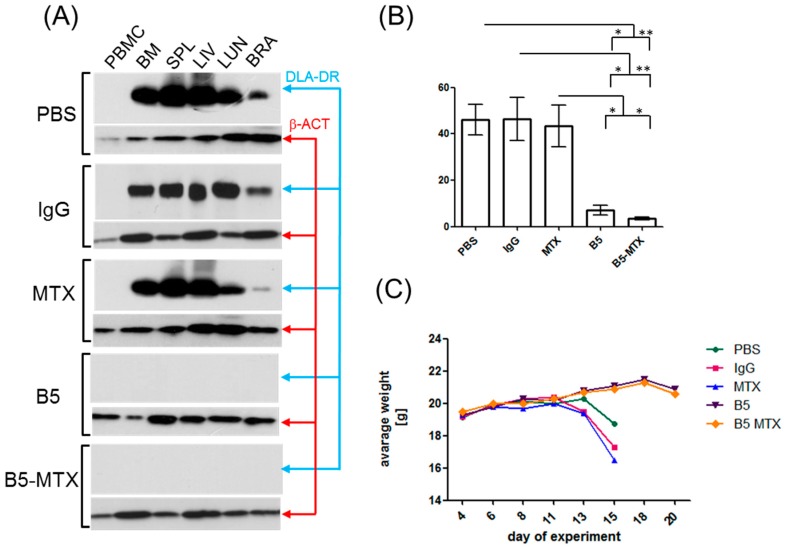
Analysis of tumor cell spread and body weight loss in CLBL-Luc tumor-bearing mice. (**A**) Protein lysates were obtained from the following organs/tissues of tumor-bearing mice: peripheral blood mononuclear cells (PBMC), bone marrow (BM), spleen (SPL), liver (LIV), lung (LUN), brain (BRA). Admixture of CLBL1-Luc cells in the above organs/tissues, reflecting tumor burden, was evaluated by Western blotting with anti-DLA-DR antibody. Protein loading was controlled with an anti-beta actin antibody (β-ACT) (**B**) Cell suspensions of bone marrows were prepared from mice treated with PBS (*n* = 8), IgG (*n* = 7), MTX (*n* = 8), B5 (*n* = 5) and B5-MTX (*n* = 5) and assessed for CLBL1-Luc cell content by flow cytometry with an anti-DLA-DR antibody, E11. (**C**) The average body weights of mice from the indicated experimental groups (*n* = 8 mice per group except for B5, *n* = 10 mice per group) were plotted. Statistically significant differences between the groups were marked with an asterisk (*** *p* < 0.001, ** *p* < 0.01).

We sought for more sensitive methods than bioluminescence to quantify tumor cell burden in bone marrows and other tissues of CLBL1-Luc-infused mice. For this purpose, Western blotting and flow cytometry with an anti-pan-DLA-DR antibody, E11 [19], was used. This antibody was chosen because it recognized a different epitope of DLA-DR than B5 and therefore did not interfere with mAbs infused for therapeutic treatment. Tumor burden in tested organs of control, but not B5- and B5-MTX-treated mice, was demonstrated by Western blotting. Specific bands corresponding to DLA-DR were present in all tested tissues except for peripheral blood mononuclear cells, and much weaker bands were found in brains (Figure 4A and Supplementary Figure S2). In bone marrows of control mice (PBS, IgG, MTX) sacrificed on day 15, CLBL1-Luc cell content exceeded 40%, but was less than 10% in the B5- and B5-MTX-treated groups (Figure 4B).

All control mice succumbed to the tumor by day 15 post CLBL1-Luc cell injection, whereas mice treated with B5 and B5-MTX experienced an >80% (22.0 ± 2.45 days versus 13.5 ± 0.86 days, *p* < 0.001) and >90% (28.0 ± 8.49 days versus 13.5 ± 0.86 days, *p* < 0.05) delay in time to tumor progression, respectively (Table 2) (TTP parameter is defined in the Materials and Methods section). B5 and B5-MTX treatment was well tolerated by the animals because neither evidence of significant weight loss resulting from off-target toxicity (Figure 4C) nor blood parameter changes (Supplementary Table S1) were noted.

### 2.4. Detection of Soluble, Circulating DLA-DR Complexes with A B5-Based Immunoassay

**Figure 5:**
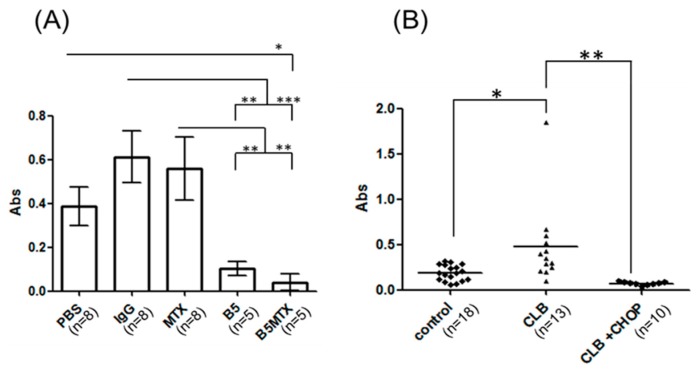
Assessment of soluble DLA-DR levels in blood sera of tumor-bearing NOD-SCID mice (**A**) and healthy dogs or canine lymphoma patients (**B**). Average serum levels (Abs) of DLA-DR in mice in the indicated experimental groups and healthy (control) or diseased dogs upon admission to the veterinary clinic (CBL) or after chemotherapy (CLB + CHOP) were plotted. Statistically significant differences between the groups were marked with asterisk * *p* < 0.05, ** *p* < 0.01, *** *p* < 0.001.

**Figure 6:**
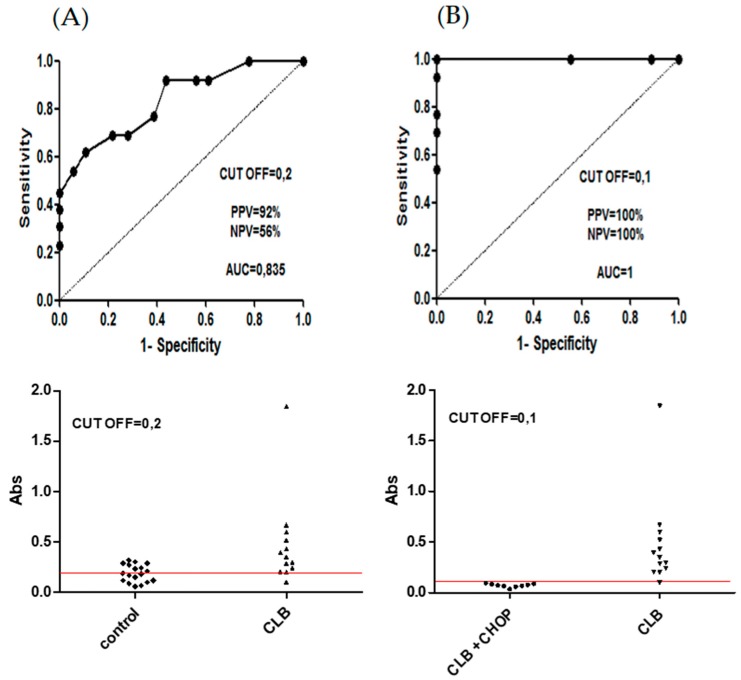
Evaluation of the diagnostic potential of soluble DLA-DR levels in canine CBL patients versus healthy control dogs (**A**), and in CBL patients versus post-chemotherapy patients (**B**). Receiver operating characteristics analysis was used to determine sensitivity, specificity, positive predictive value (PPV), negative predictive value (NPV), area under the curve (AUC) value and cut off (CUT OFF) value for the soluble DLA-DR immunoassay.

Based on a published report [25], we have hypothesized that canine B cell neoplasms can release soluble DLA-DR molecules in quantities proportional to the tumor burden. Monitoring of soluble DLA-DR levels in the blood serum of CLBL1-Luc bearing mice revealed that B5- and B5-MTX-treated groups had statistically significantly lower values than the control groups (except for the difference between the PBS and B5 groups) (Figure 5A). Therefore, we asked if differences in soluble DLA-DR levels would apply to dogs diagnosed with CBL and subjected to chemotherapy as well. To this end, the blood serum of 18 healthy control dogs, 13 dogs diagnosed with B cell lymphoma (CBL group) and 10 dogs subjected to chemotherapy during remission (CBL + CHOP) was assessed for serum sDLA-DR levels. Detailed clinical data of canine patients whose blood was used in the present study is given in Supplementary Table S2. Analysis of variance showed significant differences between the control group and the CBL group (*p* < 0.05) and between the CBL group and the CBL + CHOP group (*p* < 0.01) (Figure 5B). To further determine immunoassay performance, we analyzed sensitivity, specificity, positive predictive value (PPV) and negative predictive value (NPV) using receiver operating characteristic (ROC) analysis; two separate sets of data were analyzed. First, we sought to determine whether elevated serum sDLA-DR levels could be predictive of CBL. The results shown in Figure 6A suggest that this parameter had a strong positive predictive value of 92%, but at the same time it had a relatively low negative predictive value of 56%, and the area under the curve was 0.835. The set of data in Figure 6B was evaluated to see whether the decrease in sDLA-DR level could be used as a biomarker for successful response to chemotherapy. As shown, 100% of PPV and NPV parameters and the AUC value equal to 1 indicated that this test could reliably predict the response to chemotherapy. However, since the sample size was not pre-defined and no paired blood samples from canine patients before and after chemotherapy were available for this analysis, the above results have to be treated with caution and as preliminary.

## 3. Discussion

Targeted delivery of cytotoxic drugs using ADC technology improves their therapeutic window and minimizes chemo-associated side effects [26,27,28]. Methotrexate (MTX), a first-generation anti-folate chemotherapeutic with a narrow therapeutic window, is clinically approved for the treatment of multiple neoplasms [20,29]. It is also one of the very few chemotherapeutics with a fully known clinical profile in human and canine patients [30,31]. Relatively low potency of MTX as a payload can be ameliorated by increasing the drug to antibody ratio (DAR), while maintaining acceptable biological activity of the mAb. Reported DAR values for MTX-based ADCs depend on available lysyl and arginyl side chains in antibody molecules and range from 10 to 14.6 [23,32]. In the present work, an even higher DAR value was obtained by MTX anhydride crosslinking reaction. Despite the considerable loss of binding activity of the B5-MTX conjugate to DLA-DR, in vitro and in vivo data confirmed an increase in specific cytotoxicity against lymphoma cells expressing the target antigen. This was achieved without any substantial unspecific cytotoxicity towards the DLA-DR negative GL-1 cell line. In vivo data indicated that the B5-MTX conjugate not only showed promising anti-tumor activity in a model of advanced, disseminated lymphoma at a relatively low dose (2.5 mg/kg body weight), but had a good safety profile as well.

Signaling through anti-HLA-DR mAbs in tumor B cells activates multiple, pro-survival and pro-apoptotic pathways, but ultimately leads to cell death [12,33,34]. In the current work, we determined that apoptosis induced by B5 and B5-MTX in canine lymphoma/leukemia cell lines followed the intrinsic, caspase-dependent pathway that could partly be inhibited by ZVAD. Despite decades of clinical use, the precise mechanism of MTX cytotoxicity remains largely unknown. Available data on biological effects of MTX released from ADCs indicate a mechanism of cell sensitization [23]. Our data further support these observations, because many hallmarks of apoptotic cell death typical of B5 treatment (e.g., annexin-V binding levels and sub-G1 DNA levels shown in Supplementary Figure S1) were amplified in the case of B5-MTX.

In our hands, the CLBL1 cell line—a canine model of diffuse large B cell lymphoma (DLBCL)—was equally sensitive to the cytotoxic action of B5 and B5-MTX as the CLB70 cell line, with characteristics of chronic lymphocytic leukemia (CLL). This is in line with the observations reported by Stein et al. in models of human DLBLC and CLL cell lines treated with a humanized anti-HLA-DR antibody, IMMU-114 [12]. In this context, evolutionary conservation of death signaling pathways between human and canine hematological malignances opens interesting possibilities for comparative studies.

Both negative and positive associations of soluble HLA-DR levels in the blood serum were reported for patients with malignant melanoma and non-Hodgkin lymphoma, respectively [25,35]. Various strategies of tumor survival could account for the observed variations in sHLA-DR. We speculate that, on the one hand, melanoma cells could down-modulate HLA-DR expression in order to counteract recognition by tumor-specific effector T cells. On the other hand, a massive release of sHLA-DR by B cell neoplasms might induce tolerogenic T cell responses, which could weaken tumor immune-surveillance [24,36]. In order to assess the levels of soluble, circulating DLA-DR molecules (sDLA-DR) in the blood of tumor-bearing NOD-SCID mice undergoing experimental therapy with B5 and B5-MTX, we devised an immune-enzymatic assay based on the use of two species-specific mAbs recognizing different and non-overlapping epitopes of DLA-DR (B5 and E11) [19]. Our results strongly suggested that there was a direct correlation between tumor burden and the serum sDLA-DR levels. We could extend these observations to groups of unrelated canine patients suffering from CBL at the time of diagnosis and during remission. Although these observations offered a possibility of creating tools to help monitor the course of CBL chemotherapy in dogs, more samples, preferentially paired, from patients undergoing chemotherapy are required to fully validate this immunoassay.

## 4. Materials and Methods

### 4.1. B5-MTX Conjugate Synthesis

The B5-MTX conjugate was prepared using the method described by Goszczynski et al. [37] Briefly, 1 mg of mAb B5 in bicarbonate buffer pH 8.3 was mixed with MTX anhydride (50-molar excess). The reaction was allowed to proceed for 5 min. Next, the conjugate was separated on a Dionex Ultimate 3000 apparatus equipped (ThermoScientific, Waltham, MA, USA) with a four-component pump, autosampler with a fraction collection and a diode detector using the Superdex 200 10/300 GL resin ((GE Healthcare, Uppsala, Sweden). Isocratic elution was used with 0.1 M sodium bicarbonate at a flow rate of 0.5 mL/min. MTX concentration in conjugate was determined spectrophotometrically using detection at 280 and 372 nm as described by Ciekot, J. et al. [38].

### 4.2. Cell Lines

The CLBL1 cell line [39] was kindly provided by Dr. Barbara Rutgen (Veterinary University of Vienna, Vienna, Austria). CLB70 [40] was established by us. GL-1 [41] was kindly provided by Drs Y. Fujino and H.Tsujimoto (University of Tokyo, Tokyo, Japan). All cell lines were cultured in RPMI with 15% FBS. The stable luciferase-expressing CLBL1 cell line was generated using premade lentiviral particles (Amsbio LVP434, Abingdon, UK). Lentivirus particles were admixed with cells (1 × 10^6^/mL) at a ratio of 50 µL virus per 0.5 mL of cells. 24 h after transduction, cell culture medium was supplemented with puromycin sulfate (1.5 µg/mL). After one week of antibiotic selection, cell luminescence was validated after D-luciferin addition using a benchtop luminometer (Turner designs, TD-20/20, San Jose, CA, USA).

### 4.3. In Vitro Cytotoxicity Assays

For in vitro cytotoxicity assays, 1.25 × 10^5^ cells were seeded on a 24-well plate. The cells were preincubated for 2 h with 20 µM ZVAD. Then, 2 µg/mL of B5 or B5-MTX were added to the cells. Cytotoxicity analysis was performed after 48 h of incubation with a CellEvent™ Caspase-3/7 Green Flow Cytometry Assay Kit (Thermo Fischer Scientific, Waltham, MA, USA), according to manufacturer’s instructions. Samples were analyzed with a FACSCalibur flow cytometer (Beckton Dickinson, Franklin Lakes, NJ, USA).

To calculate IC50 values, cell lines were exposed to several concentrations of B5 and B5-MTX ranging from 0.1 to 10 μg/mL. Cell viability was determined after 48 h with propidium iodide using flow cytometry. IC50 calculation was performed with an internet tool: MLA—“Quest Graph™ IC50 Calculator.” AAT Bioquest, Inc, 25 July, 2019, https://www.aatbio.com/tools/ic50-calculator. Maximal inhibition was determined by propidium iodide staining after 24-h incubation with 10 µg/mL of B5 or B5-MTX.

### 4.4. In Vivo Monitoring of Anti-Tumor Effects of B5 and B5-MTX

NOD/SCID (NOD.CB17-Prkdcscid/NCrCrl) mice were purchased from Charles River. Mice were housed in IVC cages with a standard sterilized rodent diet and water ad libitum. All experiments using living animals were performed under permission number 117/2017 and 012/2019 from the Local Ethics Committee in Wroclaw (Poland). The anti-tumor activity of the mAb B5 and B5-MTX conjugate was assessed in vivo based on their effect on the growth of CLBL1-Luc cells transplanted intravenously into NOD/SCID mice. Forty-two mice bearing CLBL-1-Luc cells (1 × 10^7^ cells/mouse) were randomly divided into five groups (10 mice in the B5 group and eight animals in other groups). The B5-MTX conjugate, mAb B5, control, isotype matched IgG, MTX alone (0.25 mg/kg body weight) and phosphate-buffered saline (PBS) were administered intra-peritoneally on day 4 post CLBL1-Luc transplantation and repeated every two days. On day 15, all mice from the PBS, IgG and MTX groups were sacrificed because of signs of morbidity along with randomly selected five mice from the B5 group and five mice from the B5-MTX group that did not present any visible signs of disease (no weight loss nor behavioral changes). The remaining five mice from the B5 group and three mice from the B5-MTX group were treated until day 20. B5-MTX and mAb B5 were injected intraperitoneally at a dose of 2.5 mg per kg of body weight. The location of CLBL-1-Luc cells was visualized on day 14 after transplantation using an In-Vivo MS FX PRO system (Bruker INC., Billerica, MA, USA). Twenty minutes before imaging, D-luciferin potassium salt (Synchem, Felsberg, Germany) was administered to each mouse intraperitoneally at a dose of 150 mg/kg. Subsequently, animals were anesthetized with a 3% to 5% (v/v) mixture of isoflurane (Forane, Abbott Laboratories, Lake Bluff, IL, USA) in synthetic air (200 mL/min). Anesthesia was maintained by means of individual masks providing a 1.5% to 2% (v/v) mixture of isoflurane and synthetic air. Visualization was carried out using the following settings: for X-Ray t = 60 s., f-stop = 2.50, FOV = 200.0; for luminescence capture t = 4 min, binning 2 × 2, f-stop = 2.50, FOV = 200.0. Images were analyzed using Bruker MI software (Bruker INC., USA). The intensity of the luminescent signal is presented as the net intensity of the region of interest and expressed in arbitrary units [a.u.]. Time to progression (TTP) was defined as a day when either body weight loss exceeding 15% or morbidity or limb paralysis were noticed by the operator in any individual mouse.

For Western blotting analysis, mouse organs (brain, liver, lungs, bone marrow, PBMC, spleen) were suspended in a lysis buffer (20 mM Tris-HCl pH 7.5, 50 mM NaCl, 0.5% NP-40 and protease inhibitor set), and sonicated for 10 s on ice. The suspensions were centrifuged at 10,000 rpm at 4 °C for 10 min. Then, non-reducing SDS sample buffer was added to the supernatants and the samples were subjected to a 12% SDS-PAGE gel. The separated proteins were transferred onto a PVDF membrane (Millipore, Burlington, MA, USA) using semi-dry transfer. After transfer, the membrane was blocked with 1% casein in TBS at 4 °C, overnight, and subsequently incubated with 1 µg/mL primary antibody: mab E11 and anti-actin (C-4) (Santa Cruz Biotechnology, Santa Cruz, CAUSA) at room temperature for 1 h, followed by secondary horseradish peroxidase-labeled antibody (DAKO, Agilent, Santa Clara, CA, USA). Bound antibodies were visualized using the ECL blotting detection system (Thermo Fischer Scientific, USA).

### 4.5. Detection of Soluble DLA-DR Levels in the Blood Serum of Mice and Dogs

Remaining blood serum samples from 41 dogs (18 controls, 13 lymphomas and 10 during CHOP therapy), referred to the “NeoVet” veterinary clinic for periodic blood checks, and blood sera of NOD-SCID mice bearing CLBL1-Luc tumors were used for the detection of soluble DLA-DR levels. In accordance with the provisions of the Act of January 15, 2015, item 266 on the protection of animals used for scientific and educational purposes, the use of blood samples of dogs for clinical veterinary research does not require the consent of local ethics committees.

96 well plates (Nunc) were coated with B5 mAb in PBS (1 µg/mL) overnight at 4 °C. On the next day, the plates were blocked with 5% non-fat milk for 1 h at room temperature (RT), then 20 times diluted blood sera of canine tumor-bearing mice or canine patients were incubated in a 0.5% milk solution at RT for 1 h. Next, biotinylated mAb E11 (1 µg/mL) was added to the solution and incubated for 1 h at RT, followed by the incubation with the Streptavidin-HRP conjugate (1:20,000); after final washes, the 3.3′5.5′-tetramethylbenzidine substrate (Sigma, St. Louis, MO, USA) was added for a 15 to 20-min incubation. The reaction was stopped with 1 N H_2_SO_4_. The absorbance was measured at 450 nm on a Wallac Victor plate reader (Perkin Elmer, Waltham, MA, USA). Each sample was prepared in triplicate.

### 4.6. Statistical Analysis

Statistical analysis of in vivo bioluminescence imaging was performed using the non-parametric Kruskal-Wallis test, followed by Dunn’s multiple comparison tests (** *p* < 0.01, *** *p* < 0.001). Statistical analysis of all other in vitro assays was performed using one-way ANOVA with Tukey’s Multiple Comparison Test. Significance was set at * *p* < 0.05, ** *p* < 0.01, *** *p* < 0.001. Receiver operating characteristic analysis was performed by calculating the shape of the ROC curve. This was done by plotting the values of the sensitivity assay on the y-axis, and the values of false-positive rates (1-specificity) on the x-axis. The area under the curve (AUC) was subsequently calculated.

## 5. Conclusions

In this report, we tested a novel MTX-based ADC directed against canine lymphoma/leukemia cells. To our knowledge, this is the first pre-clinical study of an ADC designed for veterinary use. The results indicate a significant increase in the specific cytotoxicity of B5-MTX ADC against canine lymphoma/leukemia cell lines in comparison with unmodified mAb in vitro and in vivo. Unlike in humans, DLA-DR antigen, a target of B5 and B5-MTX, is expressed in dogs by both normal B and T cells and by mixed B/T neoplastic cells. Therefore, the use of anti-DLA-DR antibodies in diagnosis or therapy can cover up to 90% of all hematological malignancies in this species. Elevated sensitivity of canine lymphoma/leukemia cell lines to MTX opens up new opportunities for using this antimetabolite as a payload for therapeutic ADCs targeting DLA-DR. Despite the clearly lower potency of MTX in comparison to the second generation cytotoxic payload, such as auristatin, low price, simple conjugation chemistry and lack of intellectual property rights attached to this antimetabolite makes it an interesting option for veterinary use.

In this study, we have shown as well that the observed correlation of soluble DLA-DR levels in the blood serum of canine lymphoma-bearing immune-deficient mice can be further studied in the context of translation into a diagnostic test for monitoring the efficiency of chemotherapy in canine lymphoma.

## Figures and Tables

**Table 1:** In vitro cytotoxicity of mAb B5 and B5-MTX in canine lymphoma/leukemia cell lines.

Cell Line	B5		^1^ B5-MTX	
	IC_50_ nM	Maximum Inhibition (%)	IC_50_ nM	Maximum Inhibition (%)
CLBL1	9.53	69	5	85
CLB70	11.5	65	6.25	88
GL-1	n.d.	<2	n.d.	<1

^1^ ADC concentration is given as antibody concentration; n.d.—not determined.

**Table 2:** Time to tumor progression (TTP) for CLBL1-Luc bearing mice treated with B5 and B5-MTX.

Treatment	*N*	TTP (Days) ± S.D.	%PR (TF)	(*p*) B5 versus Others	(*p*) B5-MTX versus Others
B5-MTX	3	28.0 ± 8.49	100 (1)	n.s.	N.A.
B5	5	22.0 ± 2.45	100 (0)	N.A.	n.s.
MTX	8	14.0 ± 1.00	0	<0.01	n.s.
IgG	8	13.5 ± 0.86	0	<0.001	<0.05
PBS	8	14.7 ± 0.69	0	n.s.	n.s.

%PR—percentage of response to treatment, TF—number of tumor-free animals after day 40, *p*—probability, N.A.—not applicable, n.s.—not significant.

## Supplementary Materials

The following are available online at https://www.mdpi.com/2072-6694/11/10/1438/s1, Figure S1: Analysis of B5 and B5-MTX induced apoptosis in canine cell lines, Figure S2: Cytotoxicity plots used for calculating IC50 and maximum inhibition values of B5 and B5-MTX, Figure S3: Distribution of individual weight measurement of mice used for in vivo studies, Figure S4: Original scans of Western blots with densitometry analysis, Table S1: Haematological analysis of the whole blood of tumor-bearing NOD-SCID mice, Table S2: Clinical description of oncologic canine patients, Table S3: raw absorbance data of anti-sDLA-DR ELISA used for the ROC analysis.
