# Supplementary material for: An Antibody Specific for the Dog Leukocyte Antigen DR (DLA-DR) and Its Novel Methotrexate Conjugate Inhibit the Growth of Canine B Cell Lymphoma
Source: Cancers (Basel). 2019 Sep 26;11(10):1438. doi: 10.3390/cancers11101438 (PMC6827003; doi:10.3390/cancers11101438)
Supplement: Supplementary file 1 [file cancers-11-01438-s001.zip › supplementary figures.docx]

Article

An Antibody Specific for the Dog Leukocyte Antigen DR (DLA-DR) and Its Novel Methotrexate Conjugate Inhibit the Growth of Canine B Cell Lymphoma

Marta Lisowska ^1^, Magdalena Milczarek ^2^, Jarosław Ciekot ^2^, Justyna Kutkowska ^2^, Wojciech Hildebrand ^3^, Andrzej Rapak ^2,^* and Arkadiusz Miazek ^4,5,^*

**（A）**


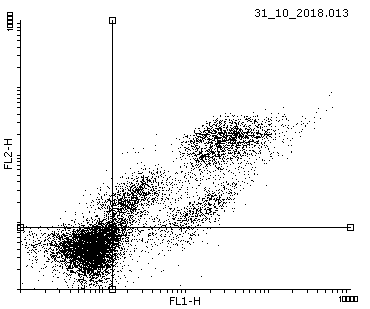

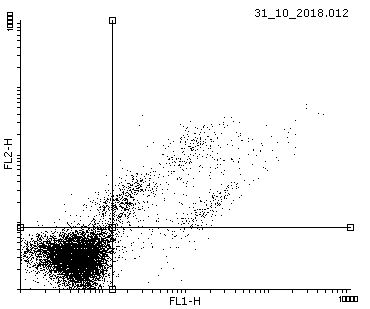

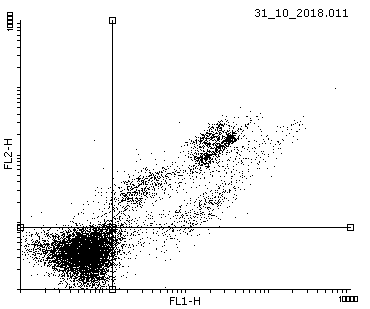

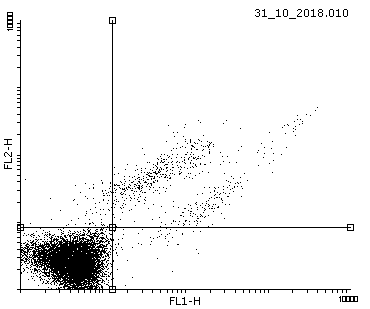


PBS

IgG

B5

B5-MTX

CLBL1

7

9

23

37

CLB70

GL1


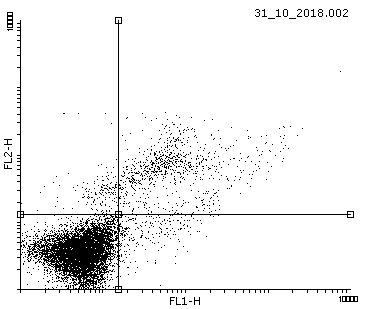

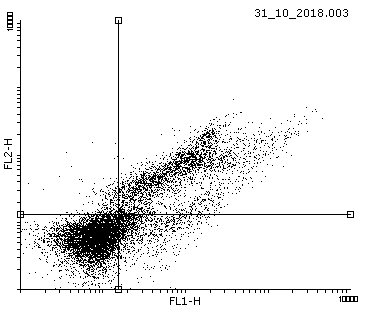

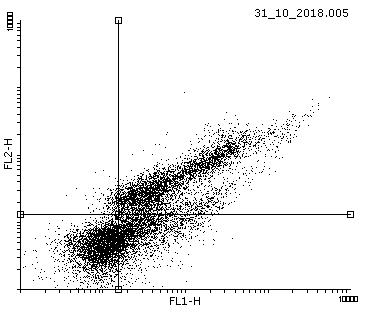

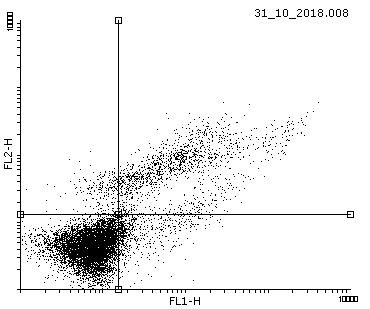

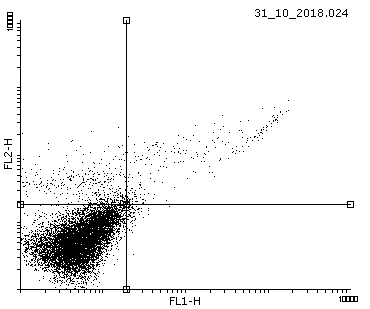

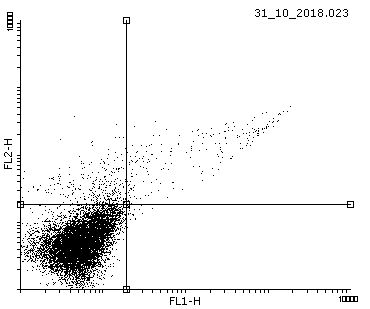

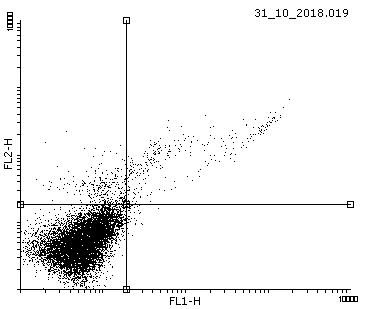

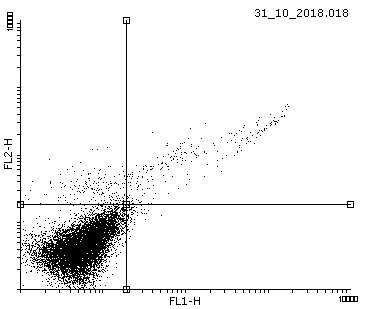


9

15

30

43

2

2

3

2

Active Caspase 3/7

SYTOX

**（B）**


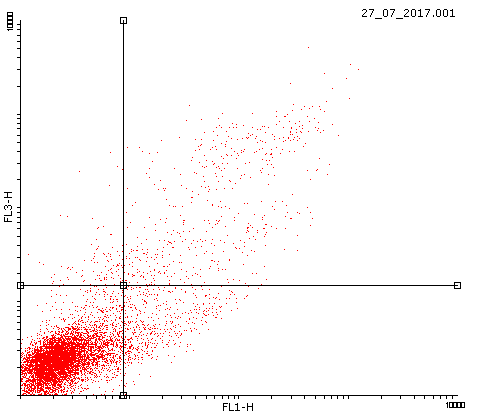

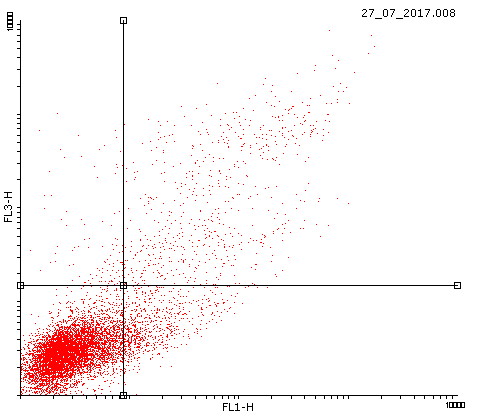

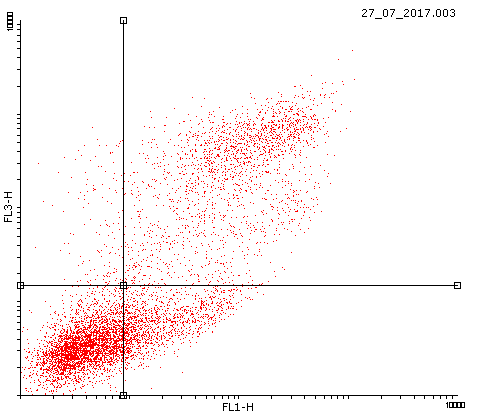

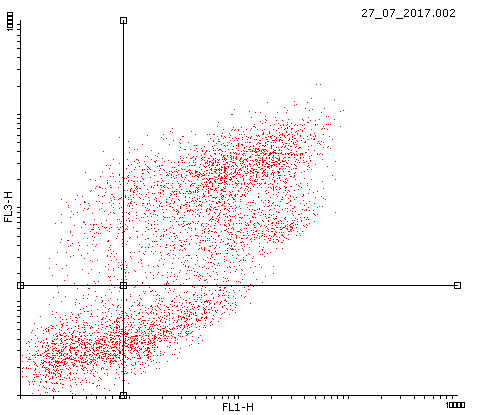


PBS

IgG

B5

B5-MTX

Annexin V

PI

CLBL1

CLB70

GL1


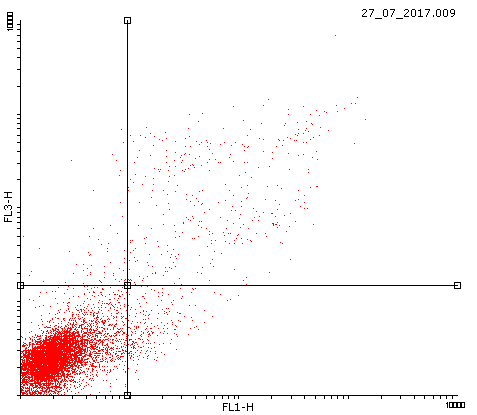

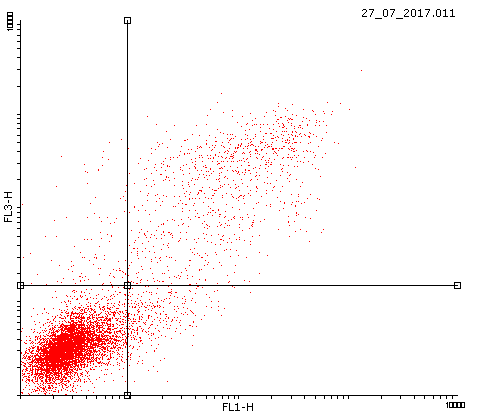

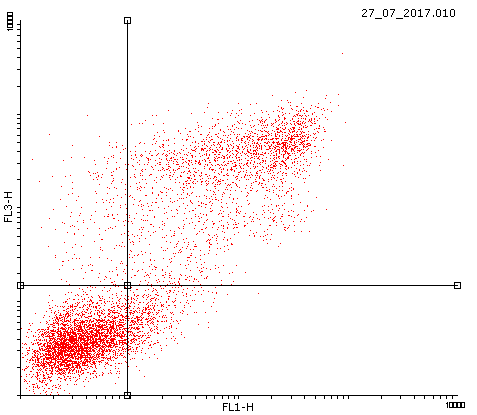

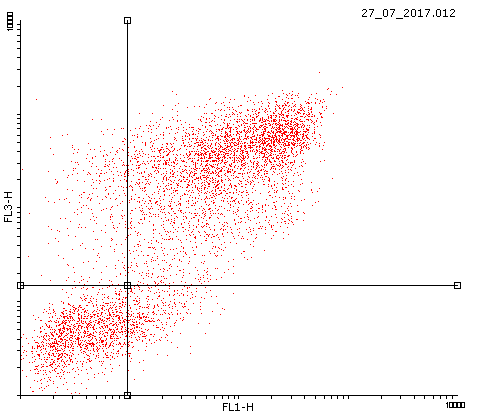

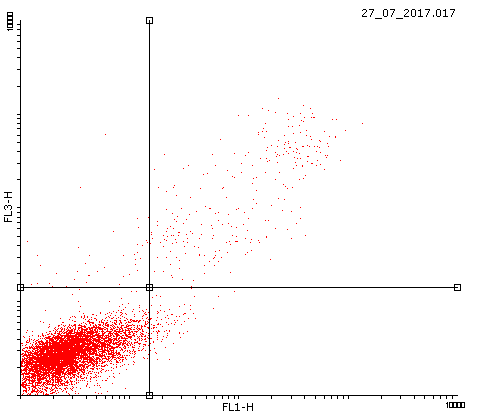

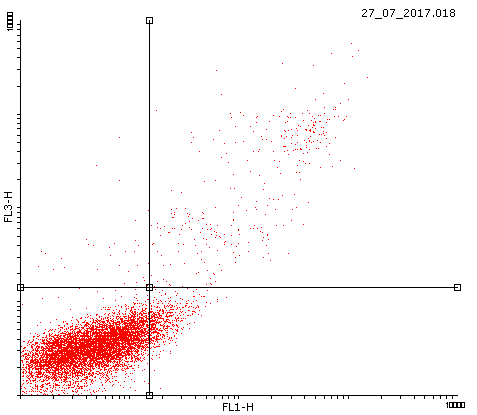

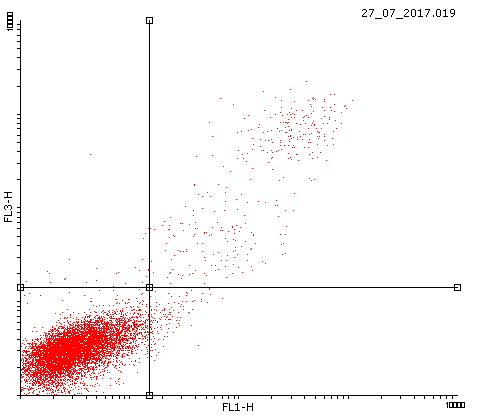

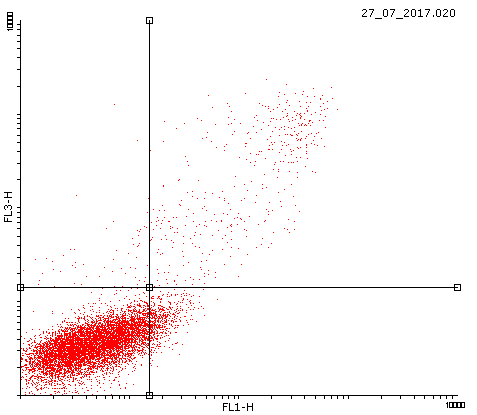


6

11

28

54

4

5

33

64

3

3

4

4

| **（C）** | **PBS** | **IgG** | **B5** | **B5-MTX** |  |
| --- | --- | --- | --- | --- | --- |
|  | 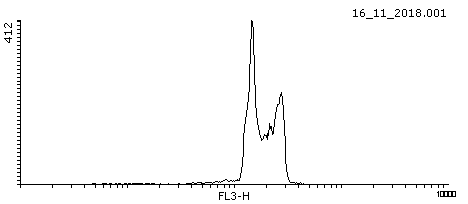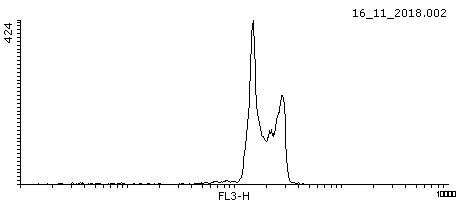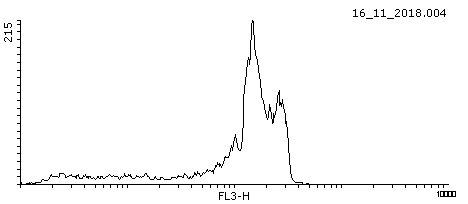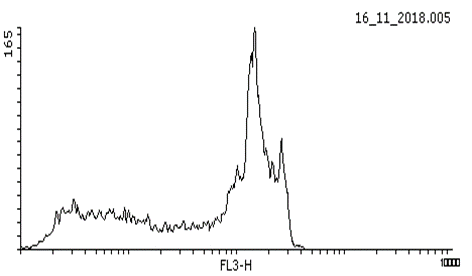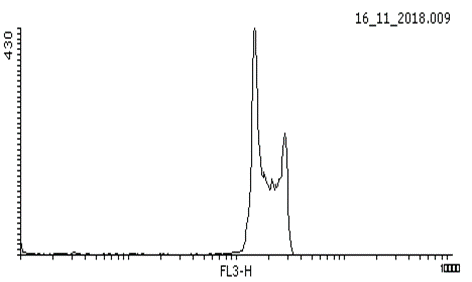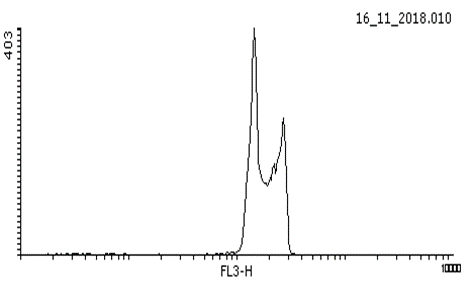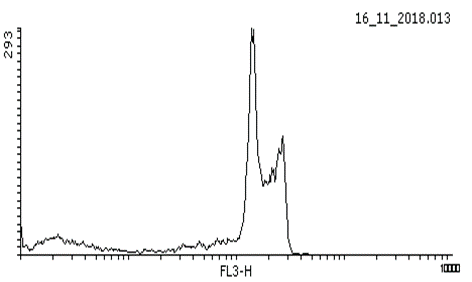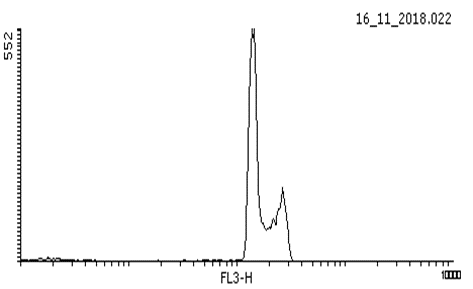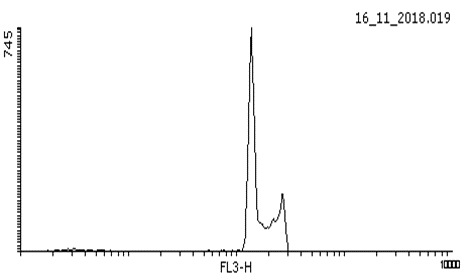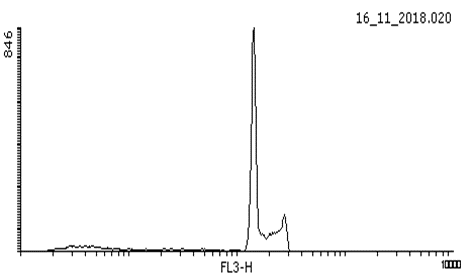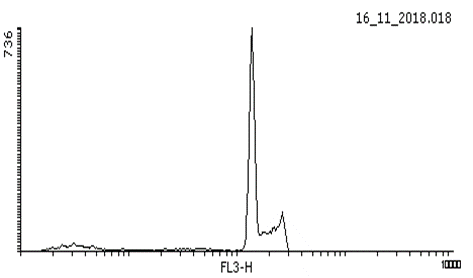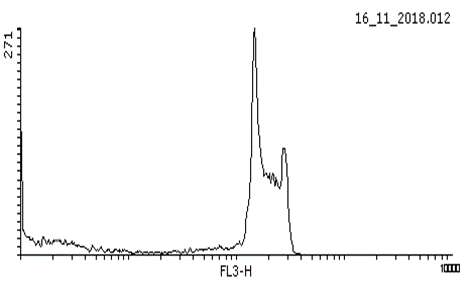 5  4  4  5  3  5  2  3  8  3  5  9 | | | | CLBL1 |
|  |  |  |  |  | CLB70 |
|  |  |  |  |  | GL1 |
|  | DNA contents | | | |  |

**Supplementary Figure S1.** Analysis of B5 and B5-MTX induced apoptosis in canine cell lines. (**A**) Caspase 3/7 activation vs cell viability (SYTOX), (**B**) Annexin-V binding vs propidium iodide staining (PI), (**C**) Sub-G1 DNA analysis was performed on indicated cell lines treated with vehicle (PBS), control IgG, B5 and B5-MTX at concentration of 2mg. Results of one experiment are shown.

| A | CLBL1  CLB70  |
| --- | --- |
| B | 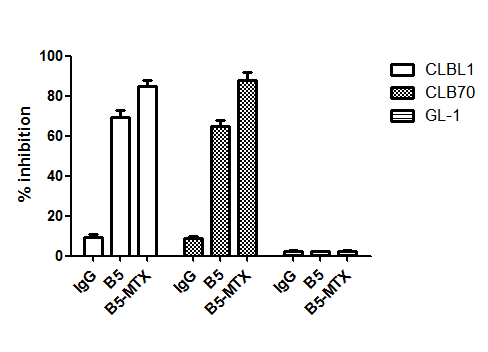 |

**Supplementary Figure S2.** (A) Mean percentages (±SD) of dead cells after treatment of CLBL1 and CLB70 cell lines with indicated molar concentrations of B5 and B5-MTX. (B) Indicated cell lines were exposed to 10mg/ml of control, isotype-matched antibody (IgG), B5 and B5-MTX for 24 hrs. Percentages of maximum cell inhibition (±SD) were calculated. and are shown in **Table 1**.

| 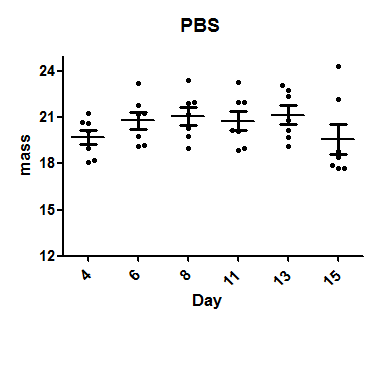 | 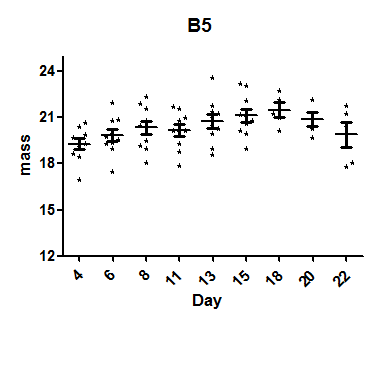 |
| --- | --- |
| 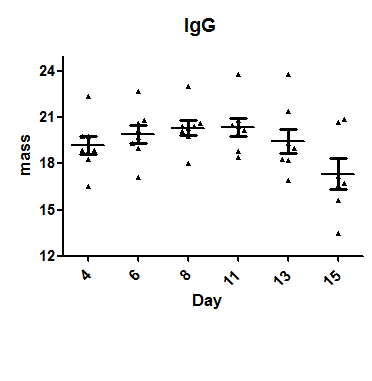 | 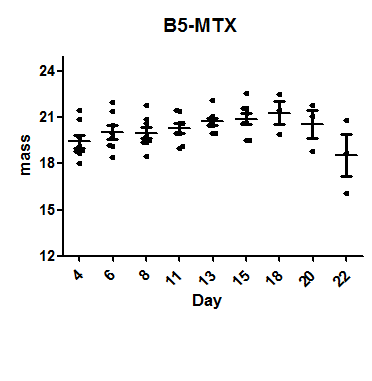 |
| 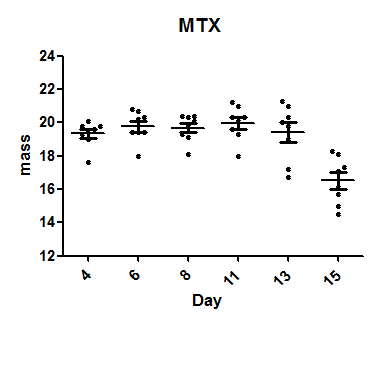 |  |

**Supplementary Figure S3.** Distribution of individual mass measurments in indicated experimental groups of mice. Mean values of these measurements were plotted in **Figure 4C.**


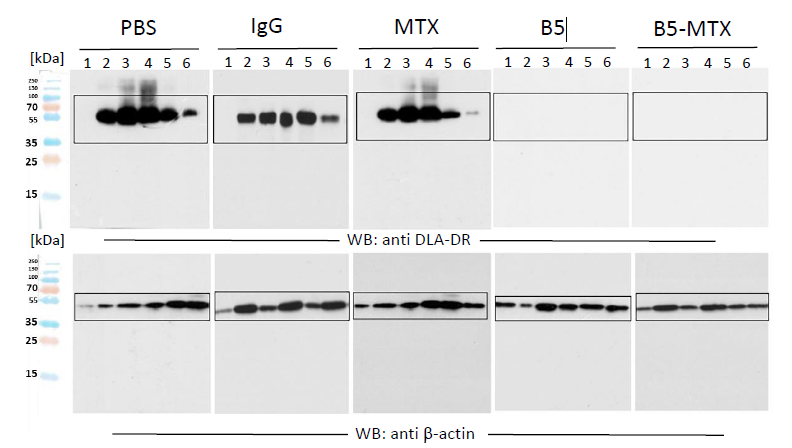


| **Lysates** | **PBS** | **IgG** | **MTX** | **B5** | **B5-MTX** |
| --- | --- | --- | --- | --- | --- |
| **1** | 0 | 0 | 0 | 0 | 0 |
| **2** | 2.78 | 0.80 | 1.49 | 0 | 0 |
| **3** | 1.74 | 2.00 | 1.93 | 0 | 0 |
| **4** | 1.57 | 0.72 | 1.44 | 0 | 0 |
| **5** | 0.59 | 2.71 | 0.44 | 0 | 0 |
| **6** | 0.22 | 0.51 | 0.21 | 0 | 0 |

**Supplementary Figure S4.** (upper panel) Original Western blot scans used in **Figure 4.**  Loading order of tissue lysates: **1**-PBMC. **2**-bone marrow. **3**-spleen. **4**-liver. **5**-lung. **6**-brain. (lower panel) Densitometry-based assessment of fold change ratio of DLA-DR protein expression in relation to β-actin expression.
